# Upper gastrointestinal pathophysiology due to mouse malaria *Plasmodium berghei* ANKA infection

**DOI:** 10.1186/s41182-019-0146-9

**Published:** 2019-03-04

**Authors:** Mizuho Shimada, Yoshie Hirose, Kazuhiko Shimizu, Daisuke S. Yamamoto, Eri H. Hayakawa, Hiroyuki Matsuoka

**Affiliations:** 1 Division of Medical Zoology, Department of Infection and Immunity, Jichi Medical University, 3311-1, Yakushiji, Shimotsuke City, Tochigi 329-0498 Japan; 2 Department of Pathology, Ashikaga Red Cross Hospital, 284-1, Yobe-cho, Ashikaga City, Tochigi 326-0843 Japan

**Keywords:** *Plasmodium berghei* ANKA, *Anopheles stephensi*, Stomach, Small intestine, Gastric red patches, Submucosal edema, Goblet cell

## Abstract

**Background:**

Epigastric pain, vomiting, and other gastrointestinal problems are among the most important symptoms of malaria infection as they suggest the possibility that the condition is serious. Pathophysiologies such as gastric mucosal changes and delayed gastric emptying have been reported in serious cases of malaria infection. However, it is unclear whether or not pathophysiological involvement of the upper gastrointestinal tract occurs in *Plasmodium berghei* ANKA (PbA)-infected mice.

**Methods:**

PbA-infective *Anopheles* mosquitoes were used to infect mice via the natural route of infection. Fifteen PbA-C57BL/6 mice were used as a cerebral malaria model and the same numbers of PbA-BALB/c mice were used as a cerebral malaria-resistant model, and then we investigated the pathophysiological involvement of the stomach and small intestine.

**Results:**

On day 8 post infection, six PbA-C57BL/6 mice showed cerebral malaria and nine others had uncomplicated infection. All the PbA-C57BL/6 mice on that same day showed severe weight loss with multiple, red gastric patches and changes to the course of the small intestine with villus goblet cell enlargement. In addition, cerebral malaria cases showed gastric gas retention with submucosal edema and small intestinal shortening. In PbA-BALB/c mice, overextension of the stomach and gas retention were evident from week 2 after PbA infection, as well as changes to the course of the small intestine and mesenteric thinning with fragility.

**Conclusions:**

We described the upper gastrointestinal pathophysiology representing new findings directly linked to malarial severity and subsequent death in PbA-infected mice as a mouse model of malaria infection.

## Background

Malaria, one of the most important mosquito-borne parasitic diseases, caused 219 million cases and 435,000 related deaths in 2017 [1]. Malarial parasites that can infect humans are classified into five species, with the most lethal among humans being *Plasmodium falciparum*. Malaria infection begins with the non-specific symptoms of acute high fever and severe headache. When it reaches the severe stage, it causes not only cerebral malaria due to sequestration of parasites in the brain [2] and brain edema [3] but also multiple organ dysfunction, including pulmonary edema and renal failure [4]. *P. falciparum* infection produces significantly more upper gastrointestinal symptoms than does *P. vivax* infection [5], and its pathophysiology may include delayed gastric emptying time, changes in the gastric mucosa, and increased gastrointestinal permeability [6–8]. Reduced fluid and food intake due to persistent upper gastrointestinal symptoms, such as epigastric pain and vomiting, may also lead to dehydration and electrolyte imbalance, which adversely affect the course of malaria infection. However, few studies have addressed the detailed course of the upper gastrointestinal pathophysiology of malaria infection in humans, and none has described the pathophysiology of the upper gastrointestinal tract, including the stomach, in mouse models of malaria.

Mice infected with *P. berghei* ANKA (PbA) [9] are widely used as a mouse model of malaria infection. PbA-infected C57BL/6 (PbA-C57BL/6) mice are the best-known cerebral malaria models [10, 11], and diagnostic imaging has revealed the presence of ischemic brain edema [12]. Intestinal floral changes and intestinal shortening have been reported as intestinal pathologies of PbA-C57BL/6 mice with cerebral symptoms [13]; however, in that study, the PbA infection route was via the infusion of infected red blood cells (RBCs) into the peritoneal cavity. In humans, inflammation of the peritoneal cavity generally causes ileus due to impaired intestinal motility, and introducing malaria-infected RBCs into the mouse peritoneal cavity may also cause intraperitoneal inflammation and affect peristalsis. In the current study, we therefore used PbA-infective *Anopheles* mosquitoes to produce PbA-infected mice, in order to investigate the pathophysiology of the stomach and small intestine in a more natural mouse model of malaria [14]. C57BL/6 mice were used as a cerebral malaria model, and BALB/c mice as a cerebral malaria-resistant model. This is the first study to describe the upper gastrointestinal pathophysiology of PbA-infected mice.

## Results

**Fig. 1:**
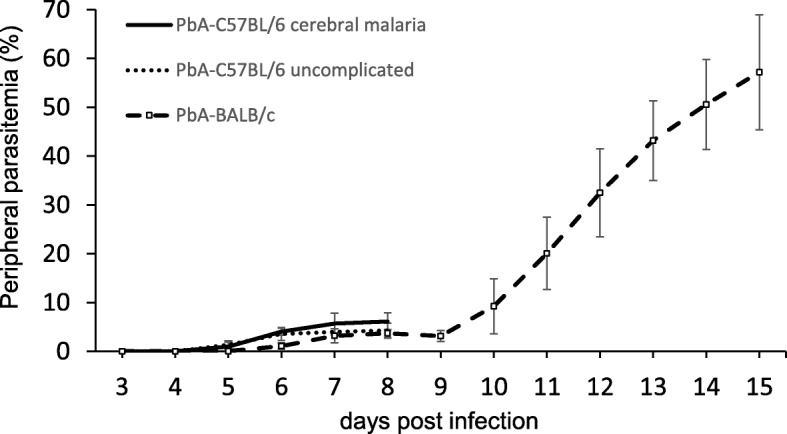
Peripheral parasitemia. Parasitemia of PbA-C57BL/6 mice with cerebral malaria (*n* = 6) and uncomplicated malaria (*n* = 9) from day 3 until day 8 post infection is shown with SD. Parasitemia of PbA-BALB/c mice from day 3 until day 15 post infection is shown with SD

We evaluated changes in peripheral parasitemia of PbA-infected mice. Among 15 PbA-C57BL/6 mice, the average parasitemia exceeded 1% on day 5 (1.2% ± 0.9%). Six PbA-C57BL/6 mice developed cerebral malaria on day 8 (four cases of convulsions and two cases of lower limb paresis), and nine other mice had uncomplicated malaria. The mice were next retrospectively divided into two groups depending on whether they showed cerebral malaria on day 8 (group A) or uncomplicated malaria on that same day (group B). In group A, peripheral parasitemia was 4.0 ± 0.7% on day 6, 5.7 ± 2.1% on day 7, and 6.1% ± 1.8% on day 8, while in group B, peripheral parasitemia was 3.5 ± 1.3% on day 6, 4.0 ± 0.6 on day 7, and 4.3 ± 1.5% on day 8 (*p* = 0.056, Student’s *t* test). In PbA-BALB/c mice, the average parasitemia exceeded 1% on day 6 (1.3% ± 1.0%) and 3.7% ± 0.8% on day 8, after which it rapidly increased to 32.5% ± 9.0% on day 12, 43.2% ± 8.1% on day 13, and 57.2% ± 11.8% on day 15 (Fig. 1). The level of peripheral parasitemia did not explain the difference between groups A and B in PbA-C57BL/6 mice.

**Fig. 2:**
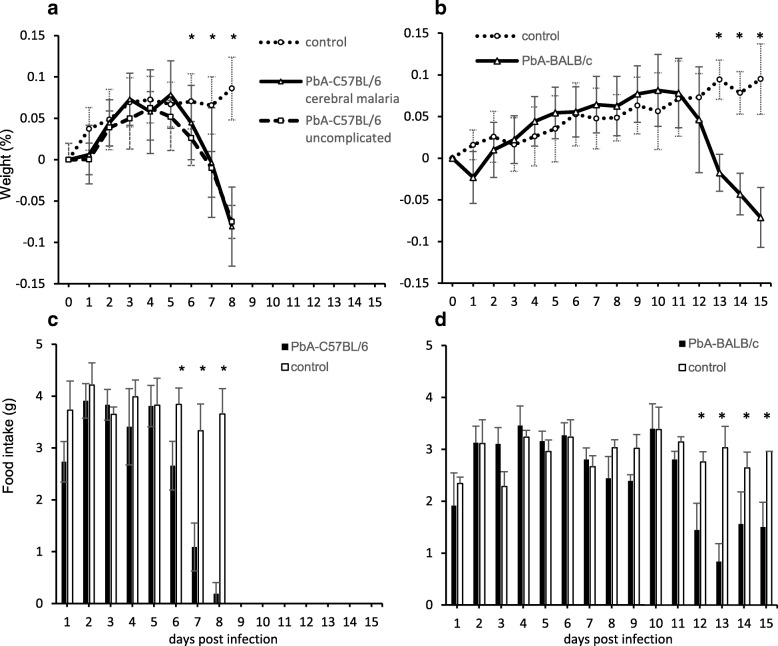
Changes in weight and food intake of PbA-infected mice. A comparison of the weight changes of PbA-C57BL/6, PbA-BALB/c, and uninfected control mice from baseline to dissection (%, mean ± SD). Food intakes in the control groups were 3.8 ± 0.2 g in uninfected C57BL/6 mice and 2.9 ± 0.3 g in uninfected BALB/c mice per day. **a** Weight changes in uninfected C57BL/6 mice and PbA-C57BL/6 mice with cerebral malaria or uncomplicated malaria until day 8 post infection. In addition to day 8, significant differences between control and PbA-C57BL/6 mice were also observed on day 6 (*p* = 0.0244, Student’s *t* test) and day 7 (*p* = 0.000576, Student’s *t* test). **b** Weight changes in uninfected BALB/c mice and PbA-BALB/c mice until day 15 post infection. In addition to days 13 and 15, significant differences between control and PbA-BALB/c mice were also observed on day 14 (*p* = 1.07 × 10^−8^, Student’s *t* test). **c** The food intake by PbA-C57BL/6 mice was decreased on day 6 (*p* = 0.000754, Mann–Whitney *U* test), on day 7 (*p* = 0.0000387, Mann–Whitney *U* test), and on day 8 (*p* = 0.00045, Mann–Whitney *U* test) compared to those in the control mice. **d** The food intake by PbA-BALB/c mice was decreased on day 12 (*p* = 0.0444, Mann–Whitney *U* test) to day 15 (*p* = 0.0444, Mann–Whitney *U* test) compared to that in the control mice. *p < 0.05

We next investigated changes in the weight and food intake of PbA-infected mice. The weight of PbA-C57BL/6 mice started to decrease significantly on day 6 post infection and was the lowest on day 8 both in groups A (− 0.08 ± 0.05% decrease) and B (− 0.08 ± 0.02% decrease), when there was a highly significant difference between the PbA-infected group and the uninfected control group (*p* = 9.27 × 10^−11^, Student’s *t* test) (Fig. 2a). The weight of the PbA-BALB/c mice started to show a significant decrease on day 13 post infection (*p* = 3.55 × 10^−7^, Student’s *t* test), and this decline was subsequently maintained (− 0.07 ± 0.04% decrease on day 15), with a highly significant difference between this group and the uninfected control group (*p* = 1.32 × 10^−9^, Student’s *t* test) (Fig. 2b). In terms of changes in weight and food intake, PbA-C57BL/6 mice (both groups A and B) virtually stopped eating on days 6, 7, and 8 post infection, with food intakes of 2.7 ± 0.5 g, 1.1 ± 0.5 g, and 0.2 ± 0.2 g, respectively (Fig. 2c), consistent with their weight loss. Food intake by PbA-BALB/c mice started to show a significant decrease on day 12 post infection (1.0 ± 0.1 g) and was subsequently maintained at 1.0 ± 0.4 g until day 15 (Fig. 2d), consistent with their sustained weight loss. PbA-infected mice thus exhibited diminished food intake and significant weight loss at specific periods after infection, depending on the strain concerned.

**Fig. 3:**
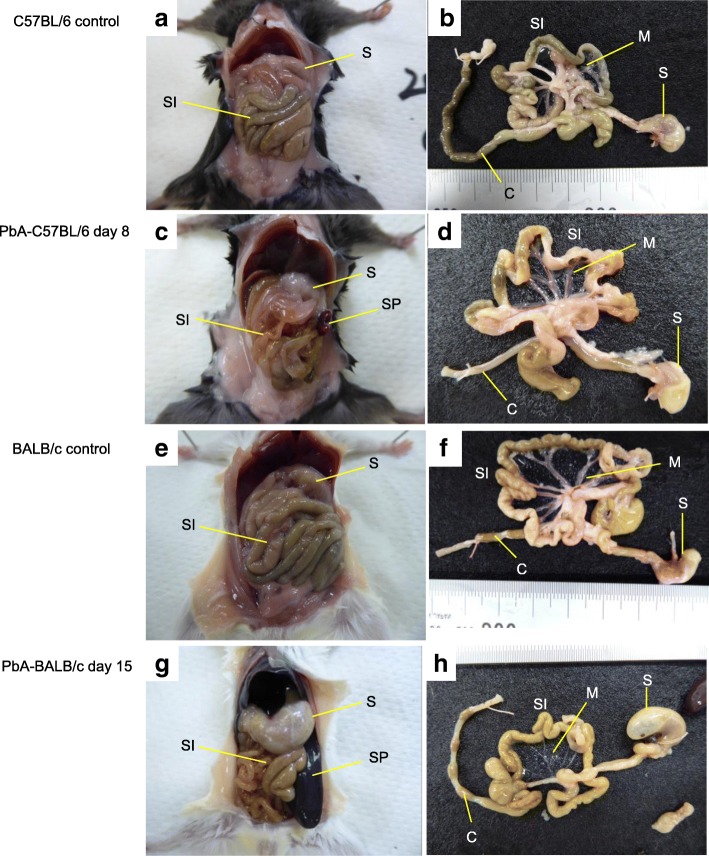
Mouse abdominal gross pathology. A PbA-C57BL/6 mouse on day 8 and a PbA-BALB/c mouse on day 15 are shown with uninfected controls. **a** Macroscopic examination of the peritoneal cavity of an uninfected C57BL/6 mouse, showing the stomach and small intestine. **b** The stomach, small intestine, mesentery, and colon after removal from the mouse shown in **a**, **c** Macroscopic examination of the peritoneal cavity of a PbA-C57BL/6 mouse revealed a change in the course of the small intestine, and the spleen started to enlarge. **d** The gastrointestinal tract after removal from the mouse shown in **c**, with an edematous small intestine with reduced intestinal content and a colon containing only a small amount of retained faces. **e** Macroscopic examination of the peritoneal cavity of an uninfected BALB/c mouse. **f** The gastrointestinal tract after removal from the mouse shown in **e**, **g** Macroscopic observation of the peritoneal cavity of a PbA-BALB/c mouse revealed splenomegaly, dilatation of the stomach, and a change in the course of the small intestine. **h** The gastrointestinal tract after removal from the mouse shown in **g**, with a thinned, fragile mesentery. C colon, M mesentery, S stomach, SI small intestine, SP spleen

We investigated the gross pathology of the gastrointestinal tract of PbA-infected mice. Six among 15 PbA-C57BL/6 mice showed cerebral malaria on day 8 (group A), and thus, all the mice in both groups A and B were dissected as with the control C57BL/6 mice when the group A mice presented cerebral symptoms. Compared with uninfected C57BL/6 mice (Fig. 3a, b), the gross pathology of group A and B mice revealed changes to the course of the small intestine (Fig. 3c). The upper small intestine was edematous, and there was reduced gastrointestinal content overall (Fig. 3d). In group A, the skulls were opened to remove the brain and examine the pathology. The brain was edematous, while bleeding or sequestration of the parasite in the bloodstream was not observed. Peritoneal examination of PbA-BALB/c mice on day 12 (six mice), on day 13 (three mice), or on day 15 (six mice) revealed not only splenomegaly, which was prominent on day 12 (3.2 ± 0.2 cm) and progressed to day 15 (3.4 ± 0.1 cm) (*p* = 0.0261, Student’s *t* test), and changes in the course of the small intestine but also gastric dilatation with much content inside. On day 15, the stomach dilatation was the most severe with excessive content and accumulated gas (Fig. 3g, h) compared with that observed in uninfected mice (Fig. 3e, f). The removed gastrointestinal tract of PbA-BALB/c mice on day 15 had an especially thin, fragile mesentery (Fig. 3h). The stomach and small intestinal involvement thus comprised changes in the course of the small intestine in both strains of PbA-infected mice. The gastrointestinal contents were reduced in all PbA-C57BL/6 mice on day 8, while stomach contents caused gastric dilatation in all PbA-BALB/c mice after day 12.

**Fig. 4:**
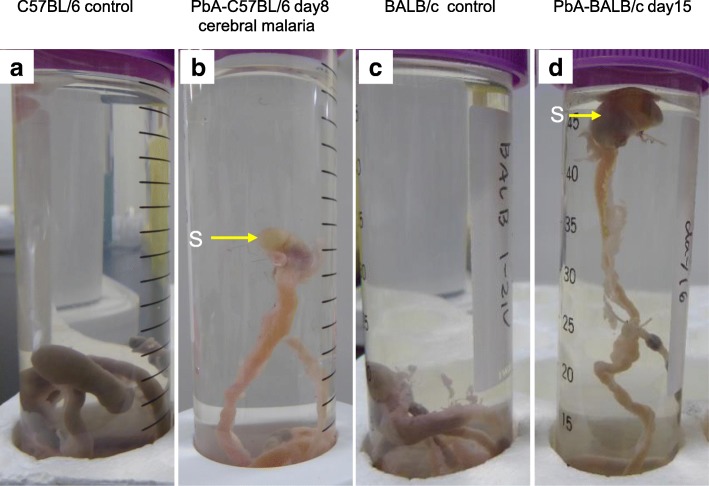
The gastrointestinal tracts in saline to examine gastric gas retention. **a** The stomach of an uninfected C57BL/6 mouse did not float. **b** The stomach of a PbA-C57BL/6 mouse with cerebral malaria floated to the middle of the tube. **c** The stomach of an uninfected BALB/c mouse did not float. **d** The stomach of a PbA-BALB/c mouse on day 15 floated to the surface. S, stomach

In PbA-infected mice, gastric gas retention was assessed by whether or not the stomach floated in saline. The absence of gas in the stomach was proved in both strains using 10 uninfected mice as controls. The stomachs of six PbA-C57BL/6 mice with cerebral malaria (group A) floated (Fig. 4b), but those of uninfected C57BL/6 mice (Fig. 4a) and nine uncomplicated PbA-C57BL/6 mice on that day (group B) did not. The stomachs of uninfected BALB/c mice (Fig 4c) and those of six PbA-BALB/c mice dissected on day 12 did not float, but mild floating was seen on day 13. Six PbA-BALB/c mice on day 15 showed severe gastric gas retention with the stomachs floating high enough to break the surface (Fig. 4d).

**Fig. 5:**
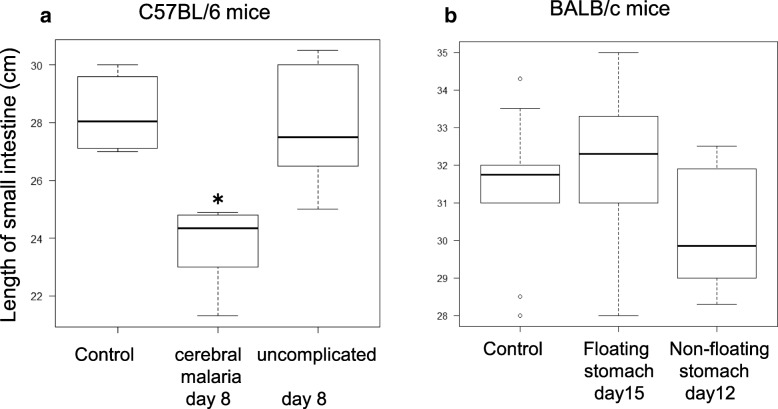
The length of the small intestine in PbA-C57BL/6 mice, PbA-BALB/c mice, and uninfected control mice. **a** Intestinal length in uninfected C57BL/6 mice (*n* = 10), PbA-C57BL/6 mice with cerebral malaria and floating stomachs (*n* = 6), and uncomplicated PbA-C57BL/6 mice with no floating stomachs (*n* = 9). **b** Comparison of intestinal length in uninfected BALB/c mice (*n* = 10), PbA-BALB/c mice with floating stomachs on day 15 (*n* = 6), and PbA-BALB/c mice without floating stomachs on day 12 (*n* = 6). *p < 0.05

We then investigated the small intestine length in PbA-infected mice by comparing the length of the small intestine in uninfected C57BL/6 mice and PbA-C57BL/6 mice in groups A (cerebral malaria) and B (uncomplicated malaria) on day 8. We found that the small intestine was only shorter in PbA-C57BL/6 mice of group A that exhibited gastric gas retention (*p* = 0.00134, Kruskal–Wallis, Steel–Dwass) (Fig. 5a). A comparison of the length of the small intestine in uninfected BALB/c mice and PbA-BALB/c mice with or without floating stomachs (six mice each on days 15 and 12) found no significant differences among any of the three groups (*p* = 0.33, Kruskal–Wallis) (Fig. 5b).

**Fig. 6:**
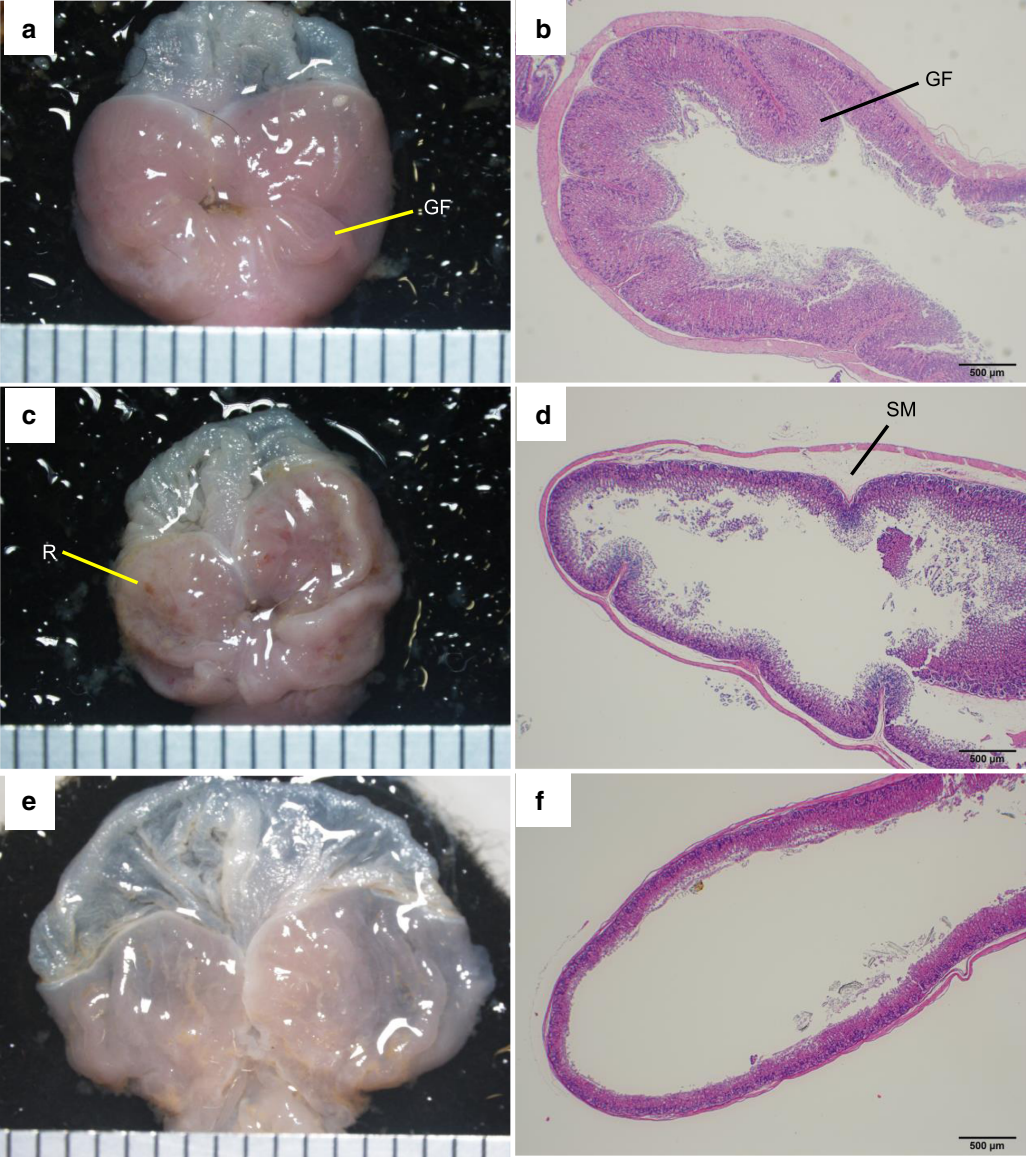
Pathology of the stomach. A PbA-C57BL/6 mouse with cerebral malaria on day 8 and a PbA-BALB/c mouse on day 15 are shown with uninfected C57BL/6 mouse. **a** Gastric mucosa of an uninfected C57BL/6 mouse, showing the gastric fold. **b** Gastric mucosa of the mouse shown in **a** stained with H&E, showing the gastric fold. **c** Multiple red patches on the gastric mucosa of a PbA-C57BL/6 mouse. **d** H&E-stained gastric mucosa of the mouse shown in **c**, exhibiting gastric submucosal edema. **e** Dilated gastric mucosa of a PbA-BALB/c mouse. **f** H&E-stained gastric mucosa of the mouse shown in **e**, showing thinning of the mucosa and the muscularis propria. GF gastric fold, R red patches, SM submucosal edema

We then evaluated the pathology of the stomach and small intestine in PbA-infected mice. Compared with uninfected mice (Fig. 6a), there were multiple red patches of gastric mucosa in both group A (cerebral malaria) and B (uncomplicated malaria) PbA-C57BL/6 mice on day 8 (Fig. 6c). Compared with the histological specimens of uninfected mice (Fig. 6b), those of PbA-C57BL/6 mice in group A displayed gastric sub-mucosal edema (Fig. 6d). In PbA-BALB/c mice, the whole stomach was distended severely on day 15 (Fig. 6e), and histopathological specimens showed severe thinning of both the mucosa and muscularis (Fig. 6f).

**Fig. 7:**
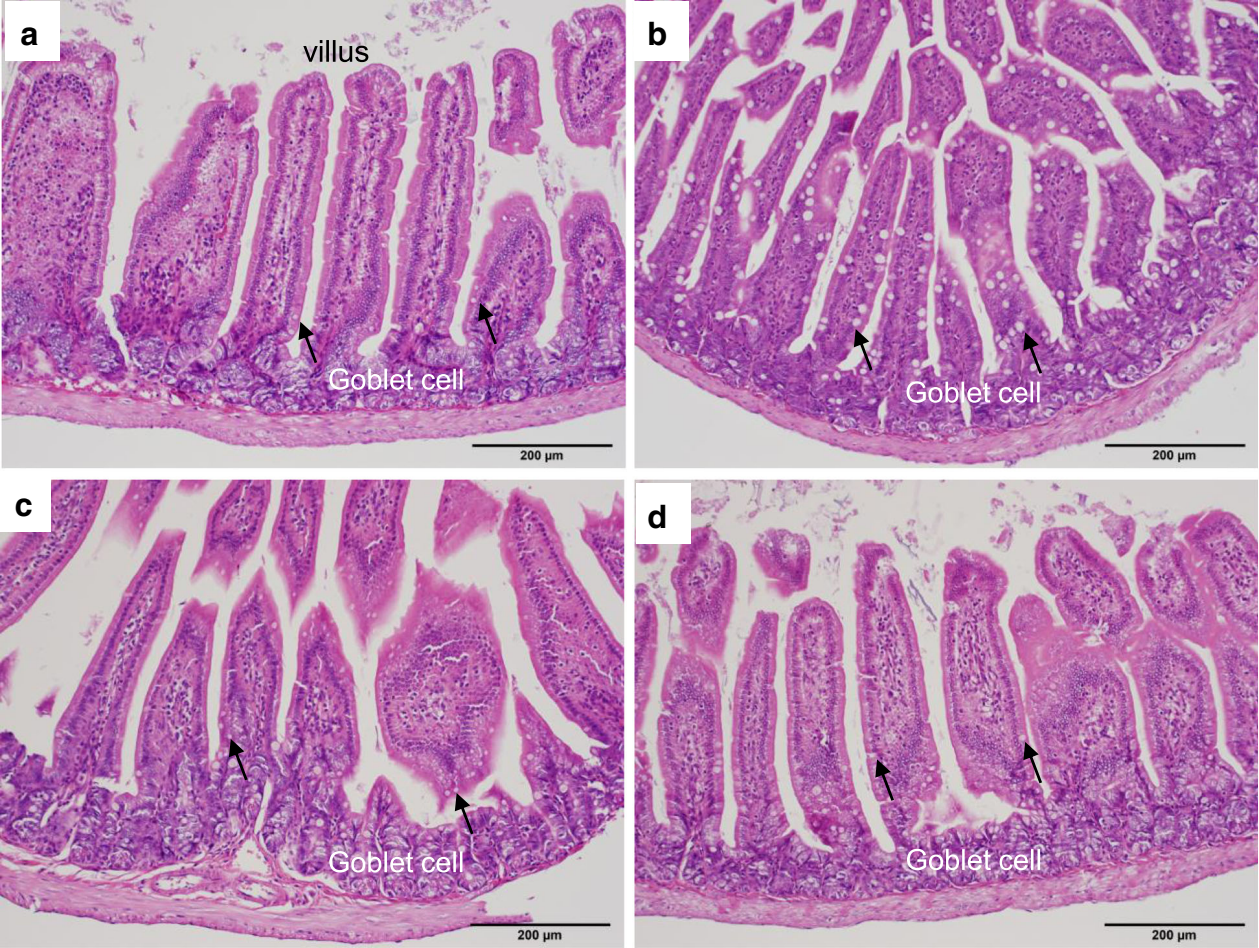
Histopathology of the small intestine. H&E-stained small intestinal villi of a PbA-C57BL/6 mouse on day 8 and a PbA-BALB/c mouse on day 15 are shown with uninfected controls. **a** The villus of a control C57BL/6 mouse. **b** Enlarged villus goblet cells of a PbA-C57BL/6 mouse. **c** The villus of a control BALB/c mouse. **d** Villus goblet cells of a PbA-BALB/c mouse

In the small intestine of PbA-C57BL/6 mice in both groups A and B, the goblet cells in villi were clearly enlarged (Fig. 7b) compared with those of uninfected C57BL/6 mice (Fig. 7a), while the goblet cells in villi of PbA-BALB/c mice were not enlarged (Fig. 7d) compared with those of uninfected BALB/c mice (Fig. 7c).

A summary of the upper gastrointestinal pathophysiology observed in PbA-infected mice is presented in Table 1.

**Table 1:** The upper gastrointestinal pathophysiology

	Stomach				Small intestine		
Mouse model	Gas retention	Dilatation	Mucosal red patches	Submucosal edema	Course change	Shortening	Goblet cell enlargement
PbA-C57BL/6 cerebral malaria day 8 (*n* = 6)	++	–	++	+	++	+	+
PbA-C57BL/6 uncomplicated malaria day 8 (*n* = 9)	–	–	+	–	++	–	+
PbA-BALB/c day 12 (*n* = 6)	–	++	–	–	+++	–	–
PbA-BALB/c day 15 (*n* = 6)	+++	+++	–	–	+++	–	–

## Discussion

We evaluated the upper gastrointestinal pathophysiology in PbA-infected mice. Overall, both PbA-C57BL/6 and PbA-BALB/c mice exhibited gastric gas retention and changes to the course of the small intestine at the time cerebral malaria developed or after a certain period post infection, but small intestinal shortening was only evident in PbA-C57BL/6 mice with cerebral malaria. In terms of pathology, PbA-C57BL/6 mice exhibited red patches in the gastric mucosa and roundly enlarged villus goblet cells in the small intestine, while the group with cerebral malaria also exhibited gastric submucosal edema. PbA-BALB/c mice developed severe gastric dilatation as well as mesenteric thinning and fragility.

Cerebral malaria was found consistently in 6/15 PbA-C57BL/6 mice on day 8 post infection via infective mosquitoes, a finding at odds with reports that cerebral malaria appeared between 6 days and 2 weeks after PbA peritoneal infection [10, 13, 15]. Although malaria infection via infective mosquito bite does not deliver a consistent number of sporozoites, intravenous infection with varying concentrations of sporozoites does not result in any difference in the prepatent period [16], and our investigation of the effect of PbA on intestinal function suggests that infection established by PbA-infective mosquitoes has the advantage of not entailing the risk of peritoneal inflammation. In the severe stage of malaria in PbA-BALB/c mice, high parasitemia is known to be completed around day 15 with severe anemia [15], leading to death in 3 weeks under an extremely high parasitemia of over 60% [13]. In this study, we found for the first time that at the point at which parasitemia rapidly increased in PbA-BALB/c mice, significant weight loss, severe gastric dilatation with gas accumulation, and mesenteric thinning with fragility occurred, and we consider that these findings are also important factors contributing to mortality.

The weight of PbA-C57BL/6 mice infected via PbA-infective mosquitoes did not start to decline immediately after infection, as is the case when PbA is delivered by peritoneal infusion [15]. Instead, weight rapidly decreased from day 6 post infection, reaching a significant reduction in food intake and weight loss by day 8. The weight of PbA-BALB/c mice exhibited a sustained decrease consistent with decreased food intake from day 12 post infection, but even on day 15, their food intake remained at around half of that of uninfected mice, consistent with the large amount of food residue and serious gastric dilatation seen in dissected PbA-BALB/c mice. Our results showed that mice infected with PbA via PbA-infective mosquitoes exhibited diminished food intake and concomitant weight loss at specific periods after infection, depending on the strain concerned.

Disease-specific gastric gas retention was evident in both strains of PbA-infected mice used in this study, but in uninfected mice, gastric contents consisting of swallowed food and air were transported to the lower gastrointestinal tract by peristaltic movements of the intestine with no excessive accumulation in the stomach. When humans develop upper gastrointestinal gas retention due to intestinal ileus, the intragastric pressure is released via a gastric tube while the underlying disease is treated. In this study, which did not include either disease treatment or intragastric decompression, gas was irreversibly retained in the stomach as the disease progressed. Studies of gastric gas retention in other species include reports of inflammatory diseases and multiple organ dysfunction in dogs [17], and this gastric gas retention was reportedly caused by the reflux of intestinal gas generated by rapidly proliferating intestinal bacteria rather than by swallowing air [18]. In mouse malaria, changes in intestinal flora have also been reported in cerebral malaria of PbA-C57BL/6 mice [13], but ours is the first study of gastric gas retention in multiple strains of PbA-infected mice. We observed gastric gas retention together with a large volume of food residue in PbA-BALB/c mice. In general, if the amount of gastric content exceeds a certain volume, this causes increased pressure in the pyloric area. This makes the difficulty in eliminating gastric contents irreversible, and a number of cases of fatal gastric distension in humans have been reported [19, 20]. In PbA-BALB/c mice, this is not inconsistent with the malaria-related deaths observed in PbA infection [13, 15]. Thus, the gastric gas retention with a large volume of food residue seen in PbA-BALB/c mice in this study is a new finding that is not inconsistent with the irreversible process leading to death.

When gastric gas retention was present, PbA-C57BL/6 mice with cerebral malaria also exhibited significant shortening of the small intestine, and this finding has also been reported in PbA-C57BL/6 mice with cerebral malaria induced by PbA peritoneal infection [13]. Small intestinal shortening is thus evident in PbA-C57BL/6 mice with cerebral malaria irrespective of the PbA infection route. However, we did not observe any significant inflammatory cell infiltration of the villous capillary vessels in infected individuals with shortened small intestines, and there were no histopathological findings that could explain the shortening seen on day 8 post infection. Intestinal length is generally considered to be influenced by smooth muscle function, and if the muscularis propria were to go into spasm in PbA-C57BL/6 mice, this might shorten the small intestine in a short period. Moreover, small intestine shortening occurred simultaneously with cerebral malaria, showing brain edema in PbA-C57BL/6 mice without bleeding or sequestration in the vessels. If small intestinal smooth muscle spasm is regarded as a sign of systemic smooth muscle spasm, it would not be inconsistent to view the cerebral malaria and small intestinal shortening as aspects of systemic smooth muscle spasm, with different symptoms appearing simultaneously at different sites.

In terms of gastric mucosal pathology, on day 8 post infection, there were multiple red patches in the gastric mucosa of PbA-C57BL/6 mice, suggesting changes to mucosal perfusion in these areas corresponding to acute gastric mucosal lesions (AGMLs) in humans. In the PbA-C57BL/6 mice that exhibited this finding, the appearance of gastric mucosal lesions may have caused the sudden decrease in food intake, causing weight loss. This gastric sub-mucosal edema was first shown in PbA-C57BL/6 mice with cerebral malaria, and in general, submucosal edema has been reported to appear in association with the severity of AGMLs [21, 22], which was concordant with our observation that edema appeared only in the cases of cerebral malaria in which gastric mucosal patches were more severe. About the involvement of the small intestine in PbA-infected mice, this is the first evidence of goblet cell enlargement in the small intestinal villi of PbA-C57BL/6 mice whether or not cerebral malaria was present, but this was not clearly evident in PbA-BALB/c mice. Goblet cells are known to produce mucin to protect against pathogens [23], and our results suggest differences between the intestinal immunity of PbA-C57BL/6 mice.

In this study, PbA infection was revealed as one of the causes of the pathophysiological involvement of the stomach and small intestine in malaria. The emergence of lesions in these organs seems to be related to the sharing of arteries, veins, and lymphatic systems with the spleen where PbA-infected RBCs cause continuous reactions to induce splenomegaly, but it is difficult to proof this at this stage.

## Conclusions

While PbA-C57BL/6 mice tend to exhibit primarily cerebral malaria, the simultaneous occurrence of pathophysiological changes in the stomach and small intestine, including changes to the course of the small intestine, small intestinal shortening with villus goblet cell enlargement, and gastric mucosal changes, represents new findings directly linked to malarial severity. We also demonstrated for the first time that the previously neglected upper gastrointestinal pathophysiology of PbA-BALB/c mice plays an important role in the irreversible severity of malaria and subsequent death.

## Methods

### Animals

Seven-week-old female mice (C57BL/6NCr and BALB/cCr) were purchased from Japan SLC. Inc. (Hamamatsu, Japan). Mice had free access to drinking water and feed purchased from CLEA Japan Inc. (CE-2; Tokyo, Japan) and were housed at a temperature of 23 °C ± 1 °C under a 12-h light-dark cycle. Fifteen mice of each strain were infected, while 10 mice were used as uninfected controls.

### Parasites and infection through infective mosquitoes

The rodent malaria parasite *Plasmodium berghei* ANKA strain clone 2.34 [24], which had been passaged in our laboratory, was used. *Anopheles stephensi* SDA 500 strain was used as a malaria transmission vector mosquito.

Infective mosquitoes were prepared in accordance with previously described procedures [25] and housed at 21 °C ± 1 °C and 80% humidity under a 12-h light-dark cycle with free access to 5% fructose with 0.05% *p*-aminobenzoic acid. Fourteen days later, 10 mosquitoes were dissected to observe the midgut. If eight or more mosquitoes developed malaria oocysts on the midgut, we used the mosquito batch for the next experiment. Eight mosquitoes from a batch were placed in a cage (16 × 26 × 26 cm) and fasted for 6 h. Then, a mouse was placed in a cage with a wire mesh and mosquitoes were allowed to bite for 4 min to complete malaria infection.

### Physical findings and anatomy

Peripheral parasitemia was measured by blood smears every day between day 3 post infection and the date of dissection. Approximately 2 μL of blood was collected from each infected mouse by making a small incision in the tail with a sterile scalpel. The blood smears were stained with Giemsa’s azur-eosin-methylene blue solution and examined under a microscope (Merck KGaA, Darmstadt, Germany), and the numbers of parasites per 3,000 RBCs were counted.

PbA-C57BL/6 mice, PbA-BALB/c mice, and uninfected mice were weighed every day from baseline to the date of dissection, and the weight on each day was divided by the weight at baseline to calculate percentage weight (%). The weights of PbA-C57BL/6 mice and uninfected C57BL/6 mice were measured until day 8, and the weights of PbA-BALB/c mice were measured until day 15. The weights of uninfected BALB/c mice were also measured until day 15. PbA-infected mice were kept in cages containing three mice each, and uninfected mice in cages containing three or four mice each. The amount of food in each cage was weighed, and the amount eaten per cage was defined as the decrease in food weight compared with that on the previous day. The daily amount eaten per mouse was estimated by dividing the amount eaten per cage by the number of mice in the cage. The cages of PbA-BALB/c mice dissected on days 12 and 13 were randomly selected.

Cerebral malaria was defined as the post-infection appearance of convulsions or paresis. All the PbA-C57BL/6 mice (*n =* 15), including the uninfected mice (*n =* 10), were dissected on the first day when cerebral malaria was detected in any of the mice. Six among 15 PbA-C57BL/6 mice showed cerebral malaria on day 8 post infection (group A) and were dissected under cerebral symptoms before death; at the same time, 9 mice with uncomplicated malaria on day 8 (group B) and 10 uninfected mice were also dissected on the same day. In group A, the skull was opened, and the pathology of the brain was observed. PbA-BALB/c mice were dissected over several days during the weight loss period, with dissection carried out on day 12 (*n* = 6), day 13 (*n* = 3), and day 15 (*n* = 6) post infection, and 10 uninfected mice were also dissected on the same day. Prior to dissection, each mouse was euthanized with carbon dioxide, after which all the blood was collected by cardiac puncture. The abdomen was then opened, the esophagus and the distal end of the colon were ligated, and the entire gastrointestinal tract was removed. The mesentery was dissected and removed en bloc with the gastrointestinal tract, and the small intestinal length and the long diameter of the spleen were measured in centimeters.

Upper gastrointestinal dysfunction was evaluated in terms of the local presence of stomach gas in the ligated and removed gastrointestinal tract. The absence of gas in the stomach was evaluated in both strains using 10 uninfected mice. The gastrointestinal tract was placed in a 50-mL centrifuge tube (Asahi Glass Co., Ltd., Japan) containing physiological saline, and gastric gas retention was assessed in terms of whether the stomach floated.

The gastrointestinal tract was then removed from the saline, an incision was made in the major curvature of the stomach, and the gastric contents were removed by rinsing with saline. After fixation with 10% formalin solution, the organs were embedded in paraffin, cut into 2-μm-thick sections, and then stained with hematoxylin and eosin (H&E).

### Microscopic observations

Stereoscopic observations were conducted with an Olympus SZX7 scope (Olympus, Tokyo, Japan), and photographs were taken with a DP73 camera (Olympus). Histopathological observations were conducted using a BX-63 microscope (Olympus), and photographs were taken with a DP72 camera (Olympus).

### Statistical analysis

Weight (in grams), peripheral parasitemia (%), and the long diameter of the spleen (in centimeters) in the two groups of mice were analyzed by Student’s *t* test. The amount of food intake was analyzed by the Mann–Whitney *U* test, and length of the small intestines (in centimeters) in the three groups of mice was analyzed by the Kruskal–Wallis test and Steel–Dwass test using EZR [26]. A *p* value less than 0.05 was considered significant.

## Abbreviations

PbA: *Plasmodium berghei* ANKA
